# PFKM-Mediated Glycolysis: A Pathway for ASIC1 to Enhance Cell Survival in the Acidic Microenvironment of Liver Cancer

**DOI:** 10.3390/biom15030356

**Published:** 2025-03-01

**Authors:** Xiaomin Wu, Boshi Wang, Yingjian Hou, Yipeng Fang, Yuan Jiang, Yuelei Song, Youyi Liu, Cheng Jin

**Affiliations:** 1 Department of Hepatobiliary Surgery, Affiliated Hospital of Jiangnan University, No. 1000 Hefeng Road, Wuxi 214041, China; 15861489930@163.com (X.W.); 6232833025@stu.jiangnan.edu.cn (Y.H.); 6232833021@stu.jiangnan.edu.cn (Y.F.); 6242833019@stu.jiangnan.edu.cn (Y.J.); 6242833002@stu.jiangnan.edu.cn (Y.S.); 2 Wuxi School of Medicine, Jiangnan University, No. 1800 Lihu Avenue, Wuxi 214122, China; 6212809066@stu.jiangnan.edu.cn

**Keywords:** hepatocellular carcinoma, ASIC1, PFKM, acidic tumor microenvironment, glycolysis, cell survival

## Abstract

The acidic tumor microenvironment plays a critical role in promoting liver cancer cell survival by enhancing glycolysis and adaptive mechanisms. Acid-sensing ion channel 1 (ASIC1) is a key regulator of pH sensing, but its role in liver cancer progression and underlying mechanisms remain unclear. In this study, we examined ASIC1 expression in clinical liver tumor tissues using immunohistochemistry and immunofluorescence, correlating it with tumor stages. HepG2 and Li-7 cells were cultured in tumor supernatant and acidic conditions to mimic the tumor microenvironment. Western blotting assessed the expression of ASIC1 and glycolysis-related enzymes, with siRNA transfections used to investigate ASIC1 and phosphofructokinase muscle-type (PFKM) in liver cancer cell survival. Our results showed that ASIC1 expression was significantly elevated in liver tumor tissues and correlated with tumor progression. Acidic conditions increased ASIC1 expression in both cell lines, enhancing cell survival, while knockdown of ASIC1 reduced viability and increased apoptosis, particularly under acidic conditions. Moreover, PFKM silencing reversed the survival advantage conferred by ASIC1, confirming PFKM as a critical downstream effector. Additionally, lactate dehydrogenase (LDH) and phosphofructokinase (PFK) activity assays showed no significant changes, suggesting other regulatory mechanisms may also be involved. These findings suggest that the ASIC1/PFKM pathway promotes liver cancer cell survival in acidic environments, representing a potential therapeutic target for disrupting tumor adaptation in liver malignancies.

## 1. Introduction

Liver cancer, including hepatocellular carcinoma (HCC) ranks as the sixth most common cancer and is the fourth leading cause of cancer-related deaths worldwide [1]. Several risk factors are associated with liver cancer development, including chronic hepatitis B (HBV) or hepatitis C (HCV) infection, aflatoxin exposure, excessive alcohol intake, obesity, type 2 diabetes, and smoking [2]. Current treatment options for liver cancer encompass surgical interventions (hepatic resection and liver transplantation), image-guided ablation, radiotherapy, transarterial therapies, and systemic treatments [3]. However, as the majority of liver cancer cases are diagnosed at advanced stages, therapeutic outcomes remain unsatisfactory. Therefore, it is critical to elucidate the molecular mechanisms underlying liver cancer and to identify more effective therapeutic strategies.

A key feature of solid tumors, including liver cancer, is their acidic microenvironment [4]. According to the Warburg effect, solid tumors preferentially utilize glycolysis for ATP production, even in the presence of adequate oxygen, instead of oxidative phosphorylation [5,6,7]. This metabolic reprogramming contributes to a range of tumor-promoting features, including rapid proliferation, immune evasion, and enhanced invasiveness. It also leads to the accumulation of glycolytic byproducts, such as lactic acid and pyruvic acid, contributing to the acidification of the tumor microenvironment [8,9]. In the context of liver cancer, recent studies emphasize the importance of targeting the acidic niche as a therapeutic strategy, as this could potentially disrupt metabolic advantages tumor cells gain through the Warburg effect [10,11]. One critical enzyme in glycolysis is 6-phosphofructokinase 1 (PFK1), which catalyzes the conversion of fructose-6-phosphate to fructose-1,6-bisphosphate (F-1,6-BP) [12]. Previous studies have shown that PFK1 enhances glycolysis, driving cell proliferation and tumor growth in various cancers, such as glioblastoma and colon cancer [13,14,15]. PFK1 exists in three isoforms: PFKM, PFKL, and PFKP, with PFKM, encoded by the *PFKM* gene, playing a crucial role in muscle glycolysis and gaining recognition for its role in cancer progression [16]. Notably, ZEB1 has been reported to upregulate PFKM, promoting glycolysis and thus advancing liver cancer progression and metastasis [17]. Nonetheless, the exact role of PFKM in mediating cell survival within an acidic tumor microenvironment requires further investigation.

Acid-sensing ion channels (ASICs) are a subfamily of the degenerin/epithelial sodium channel (DEG/ENaC) superfamily [18]. The ASIC family consists of several subunits, including ASIC1, ASIC2, ASIC3, and ASIC4 [19]. Initially, research focused on ASIC1 due to its widespread expression in the central and peripheral nervous systems [20]. Recent evidence supports that ASIC1 also participates in promoting tumor cell proliferation, migration, and survival in acidic environments [21]. For instance, ASIC1 has been shown to promote HCC cell migration and invasion by inducing autophagy in response to acidosis [22]. However, its precise mechanisms for promoting survival under low pH conditions in liver cancer remain poorly understood. Although HepG2 cells were historically derived from hepatoblastoma, they are extensively used in liver tumor research, especially in metabolism and acidification-related studies. To strengthen the validity of our conclusions, we conducted parallel experiments in a second liver cancer cell line, Li-7. In this study, we investigated the expression of ASIC1 in clinical liver tumor tissues, including its correlation with serum alpha-fetoprotein (AFP) levels, and explored its mechanistic role in promoting cell survival under acidic conditions. Our findings suggest that ASIC1 enhances liver cancer cell survival by upregulating PFKM-mediated glycolysis in an acidic tumor microenvironment. These results reveal ASIC1/PFKM as a potential therapeutic target for liver malignancies.

## 2. Materials and Methods

### 2.1. Clinical Samples

This study was conducted in accordance with ethical standards and was approved by the Human Research Ethics Committee of the Affiliated Hospital of Jiangnan University (approval number: LS2022068). Written informed consent was obtained from all participants prior to their inclusion in the study. A total of 66 paired liver tumor and adjacent non-tumor tissue samples were collected from patients who had undergone primary surgical resection. Immediately following resection, tissue samples were snap-frozen in liquid nitrogen and stored at −80 °C until further analysis. Tumor stages were determined using the TNM staging system from the 7th edition of the American Joint Committee on Cancer (AJCC) (Table S1). To investigate the relationship between ASIC1 and AFP, we performed a Spearman correlation analysis using the TCGA-LIHC dataset.

### 2.2. Immunofluorescence

Formalin-fixed and paraffin-embedded tissue sections (5 μm) were deparaffinized and rehydrated through a graded series of ethanol. Antigen retrieval was performed by heating the sections in citrate buffer (pH 6.0) for 10 min in a microwave. After cooling, sections were washed with phosphate-buffered saline (PBS) and then blocked with 5% bovine serum albumin (BSA) and 0.3% Triton X-100 (ST797, Beyotime, Shanghai, China) for 1 h at room temperature to prevent non-specific binding. The sections were incubated overnight at 4 °C with primary antibodies specific to human ASIC1 or other relevant markers. After washing with PBS, tissue sections were incubated with a secondary antibody conjugated to Alexa Fluor 488 (A11008, Invitrogen, CA, USA) for 1 h at room temperature in the dark. Following counterstaining with DAPI to visualize nuclei, the sections were mounted using antifade mounting medium. Fluorescence images were captured using a ZEISS LSM880 confocal microscope (Axio Vert A1, ZEISS, Germany), and the expression of ASIC1 was quantified by analyzing fluorescence intensity with Image J (v1.5.3) software.

### 2.3. Immunohistochemistry

Formalin-fixed and paraffin-embedded tissue samples were sectioned into 5 μm thick slices. Sections were incubated overnight at 4 °C with a primary human anti-ASIC1 antibody. A biotinylated secondary antibody, followed by streptavidin–horseradish peroxidase (HRP) conjugation, was applied using a DAB (3,3′-diaminobenzidine) staining kit according to the manufacturer’s protocol. Cells displaying brownish-yellow staining in the membrane or cytoplasm were considered positive for ASIC1. Stained tissue sections were captured digitally at 400× magnification using an Pannoramic MIDI (3DHISTECH Ltd., Hungary). The intensity and percentage of ASIC1-positive cells were quantified using Image J software.

### 2.4. Cell Culture

HepG2 and Li-7 cells were provided by the Stem Cell Bank of the Chinese Academy of Sciences, Shanghai, China. Both cell lines were authenticated using short tandem repeat (STR) profiling and passaged for fewer than six months after receipt. Cells were cultured in Dulbecco’s Modified Eagle Medium (DMEM; Gibco, CA, USA) supplemented with 10% fetal bovine serum (FBS; Gibco, CA, USA), penicillin (100 U/mL), and streptomycin (100 µg/mL). Cells were maintained in a humidified incubator with 5% CO_2_ at 37 °C. To model the acidic tumor microenvironment, we adjusted the medium to pH 7.4, pH 6.5, or pH 6.0 using 1 N HCl and HEPES buffering. We measured the pH at the start and end of a 48 h culture period, noting only minor fluctuations (±0.1–0.2 units). This approach allowed us to maintain pH 6.5 as a moderately acidic condition, consistent with reported tumor pH values (~6.8–6.9), and pH 6.0 to represent more severe acidosis.

### 2.5. Preparation of Culture Supernatants from HepG2 Cell Lines

HepG2 cells (5 × 10^6^) were seeded in 10 mL of complete DMEM in 100 mm culture dishes. After 48 h, the culture supernatants were harvested and centrifuged at 450× *g* for 10 min to remove debris, followed by a second centrifugation at 1500× *g* for 15 min. The tumor supernatant (TSN) medium was prepared by mixing the HepG2 supernatant with fresh normal culture medium at a 1:1 ratio. Aliquots of the TSN were stored at −80 °C until use.

### 2.6. Plasmids or siRNA Transfection

For PFKM knockdown, small interfering RNA (siRNA) specific to PFKM and control siRNA were obtained from Genechem (Table S2) (Shanghai, China). Transfections were performed following the manufacturer’s protocol. The PFKM siRNA sequences were designed to target the coding region of PFKM mRNA, and the most effective siRNA for PFKM inhibition was selected based on screening through Western blot analysis.

For ASIC1 knockdown, siRNA transfection was performed using previously validated siRNA sequences, as referenced in our previous study [23]. All siRNA transfections were carried out using Lipofectamine 2000 (Invitrogen, CA, USA) according to the manufacturer’s instructions. Cells were incubated for 48 h post-transfection before subsequent experiments. Knockdown efficiency was confirmed by Western blotting.

### 2.7. Western Blot Analysis

Protein extraction was performed using lysis buffer containing protease inhibitors. Protein samples were separated on 8% or 10% sodium dodecyl sulfate–polyacrylamide gel electrophoresis (SDS-PAGE) gels and transferred onto polyvinylidene difluoride (PVDF) membranes. Membranes were blocked with 5% nonfat milk in Tris-buffered saline with 0.1% Tween-20 (TBST) for 2 h at room temperature. Primary antibodies, including anti-ASIC1, anti-HK2, anti-PFKM, anti-PKM2, and anti-β-actin (all from Proteintech, Wuhan, China), were incubated overnight at 4 °C. After washing, the membranes were incubated with corresponding HRP-conjugated secondary antibodies. Protein expression was detected using enhanced chemiluminescence reagents, and the density of protein bands was quantified using ImageJ software.

### 2.8. Cell Counting Kit-8 (CCK-8) Assay

HepG2 cells (3000 per well) were seeded into 96-well plates and incubated at 37 °C in a 5% CO_2_ humidified atmosphere. After treatments, 10 μL of CCK-8 reagent (CA1210, Solarbio, Beijing, China) was added to each well and incubated for 1 h at 37 °C. Absorbance was measured at 450 nm using a microplate reader (Thermo Fisher Scientific, Waltham, MA, USA) to assess cell viability.

### 2.9. Apoptosis Assays

HepG2 cells transfected with either ASIC1 overexpression plasmids, siRNA-ASIC1, or siRNA-PFKM were seeded into 12-well plates and incubated in a humidified atmosphere with 5% CO_2_ at 37 °C. Cells were then cultured in TSN pH 6.0 or normal medium pH 6.0. After 12 h in suspension, apoptosis was evaluated using an Annexin V-FITC/propidium iodide (PI) apoptosis detection kit (BD Biosciences, NJ, USA) according to the manufacturer’s instructions. Apoptotic cells were quantified using flow cytometry.

### 2.10. LDH and PFK Activity Assays

Lactate dehydrogenase (LDH) activity was measured using a commercial kit from Beyotime (catalog no. P0395S, Shanghai, China), while phosphofructokinase (PFK) activity was determined with a kit from Solarbio (catalog no. BC0535, Beijing, China). Each assay was performed strictly according to the manufacturer’s instructions. All results from these assays are also presented in the Supplementary Materials.

### 2.11. Statistical Analysis

All experimental data were presented as mean ± standard deviation (SD) from at least three independent experiments. Statistical significance was evaluated using Student’s *t*-test or one-way analysis of variance (ANOVA) followed by post hoc tests. A *p* value of less than 0.05 was considered statistically significant. *p*-values are presented as “*, *p* < 0.05”, “**, *p* < 0.01”, “***, *p* < 0.001”, or “****, *p* < 0.0001” in the figures and text. Statistical analyses were performed using GraphPad Prism software (v9).

## 3. Results

### 3.1. Characterization of ASIC1 Expression in Liver Tumor Tissues

**Figure 1:**
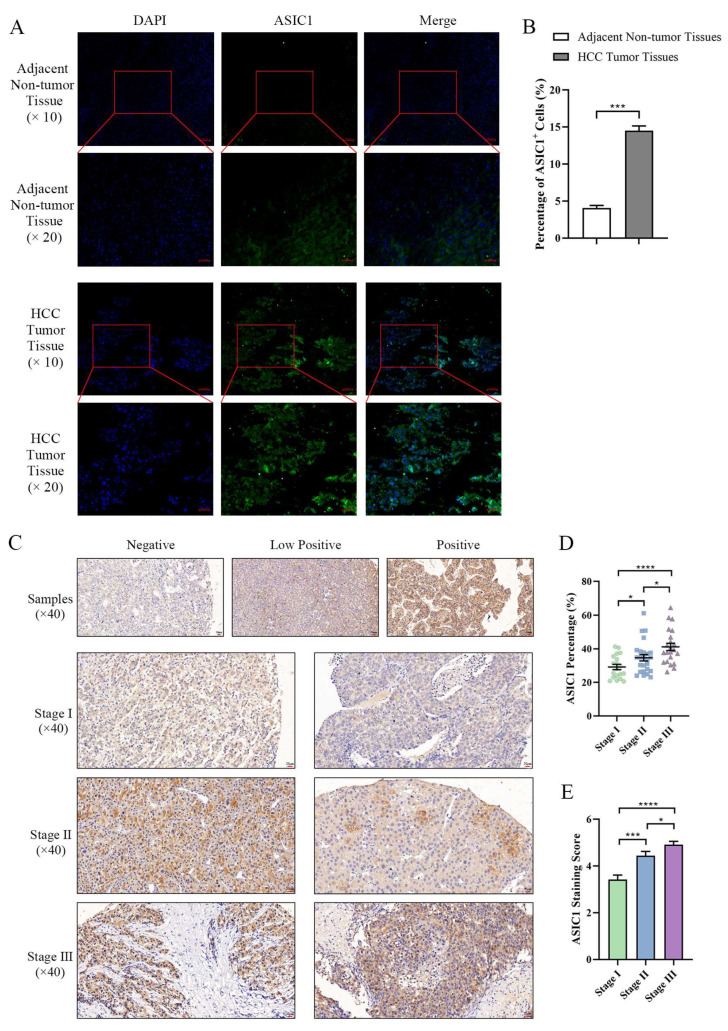
ASIC1 expression is upregulated in liver tumor tissues and correlates with tumor stage. (**A**) Immunofluorescence staining shows ASIC1 expression in HCC tumor tissues and adjacent non-tumor tissues. Representative images are presented at 200× and 100× magnification. (**B**) Quantification of ASIC1-positive cells in tumor and adjacent non-tumor tissues. (**C**–**E**) Immunohistochemical staining of ASIC1 in liver tumor tissues at different clinical stages (Stage I, Stage II, and Stage III), showing a progressive increase in ASIC1 expression with advancing tumor stage. Representative images and quantification of ASIC1 expression are presented. The graphs show the means ± SEM of at least three independent experiments, **** *p* < 0.0001, *** *p* < 0.001,* *p* < 0.05.

Our immunofluorescence analysis of post-surgical liver tumor tissue samples revealed that ASIC1 expression was significantly upregulated in tumor tissues compared to adjacent non-tumor tissues (Figure 1A,B). To further characterize ASIC1 expression patterns, we stratified liver tumor tissues based on clinical and pathological parameters. According to the 7th edition of the American Joint Committee on Cancer (AJCC) staging system for liver cancer, the samples were classified into Stage I (19 cases), Stage II (25 cases), and Stage III (22 cases). Immunohistochemical (IHC) staining demonstrated a progressive increase in ASIC1 expression across advancing tumor stages, with significantly higher ASIC1 levels observed in Stage III compared to earlier stages (Figure 1C–E). These findings indicate that ASIC1 upregulation may be associated with liver cancer progression.

We additionally examined the correlation between ASIC1 and AFP levels using the TCGA-LIHC dataset. Spearman correlation analysis revealed a weak but statistically significant positive correlation (r = 0.25, *p* < 0.001, Figure S1 and Table S3). Interestingly, subgroup analyses showed that this correlation was more evident in the low ASIC1 expression group (r = 0.20, *p* = 0.04) and became non-significant in the high ASIC1 expression group (r = 0.06, *p* = 0.55). These results suggest that ASIC1 may influence AFP expression, particularly at lower expression ranges, although additional research is needed to clarify the underlying mechanisms.

### 3.2. Tumor Acidic Microenvironment Drives ASIC1 Expression and Cell Survival in Liver Cancer Cells

**Figure 2:**
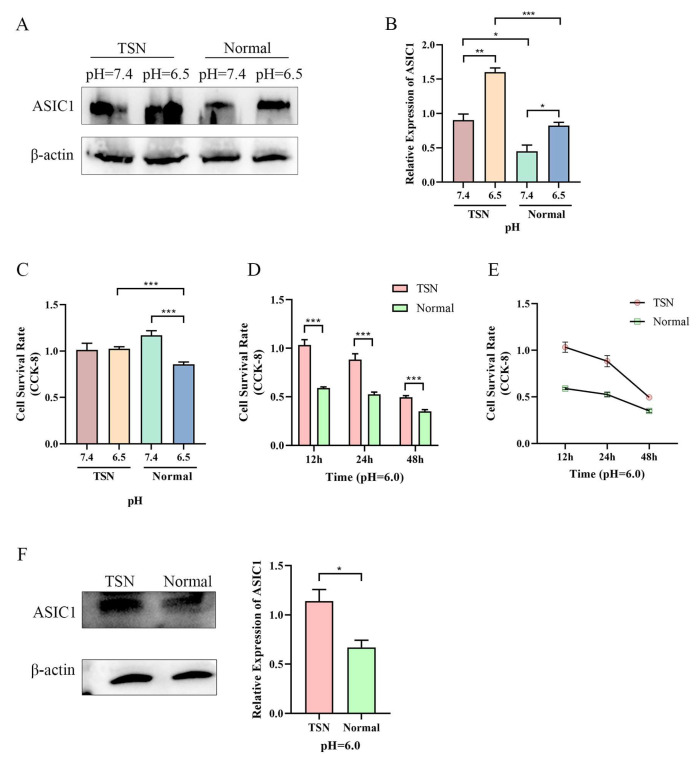
The tumor acidic microenvironment induces ASIC1 expression and promotes HepG2 cell survival. (**A**,**B**) Western blot analysis of ASIC1 expression in HepG2 cells cultured in tumor culture supernatant (TSN) and normal medium (Normal) at pH 7.4 and pH 6.5, showing upregulation of ASIC1 in acidic conditions. (**C**) CCK-8 assay demonstrates reduced HepG2 cell viability at pH 6.5 compared to pH 7.4, but cells in TSN maintain higher viability than those in normal medium. (**D**,**E**) CCK-8 assay shows higher cell viability in TSN at pH 6.0 compared to normal medium at pH 6.0. (**F**) Western blot confirms increased ASIC1 expression in TSN at pH 6.0 compared to normal medium. Data are expressed as the mean ± SEM from at least three independent experiments, *** *p* < 0.001, ** *p* < 0.01, * *p* < 0.05. Original images of (**A**,**F**) can be found in Supplementary Materials.

To model an acidic tumor microenvironment, HepG2 cells were cultured in tumor supernatant (TSN) or normal medium at pH 7.4 and pH 6.5. Western blot analysis showed that ASIC1 expression increased at pH 6.5 in both normal medium and TSN (Figure 2A,B). Of note, the pH 6.5 condition represents a moderately acidic environment, close to the in vivo tumor pH (~6.8–6.9). Cell viability decreased in the normal medium but remained higher in TSN, suggesting that additional factors in TSN support cell survival (Figure 2C).

At pH 6.0, a more severe acidosis, cell viability in the normal medium was substantially reduced, whereas viability in TSN was preserved to a greater extent (Figure 2D,E). ASIC1 expression was strongly induced under pH 6.0 TSN (Figure 2F), indicating that ASIC1 may be critical for cell survival in severe acidity. While ASIC1 expression indeed increased in response to acidity, the net effects on viability also depended on the presence of other survival factors in TSN. In the normal medium, moderate ASIC1 upregulation at pH 6.5 was insufficient to counteract acid-induced cell damage, resulting in an overall reduction in viability. In contrast, cells grown in TSN likely benefited from additional tumor-secreted factors that enhanced survival under acidic conditions.

### 3.3. ASIC1 Promotes Cell Survival in Tumor Acidic Microenvironment

**Figure 3:**
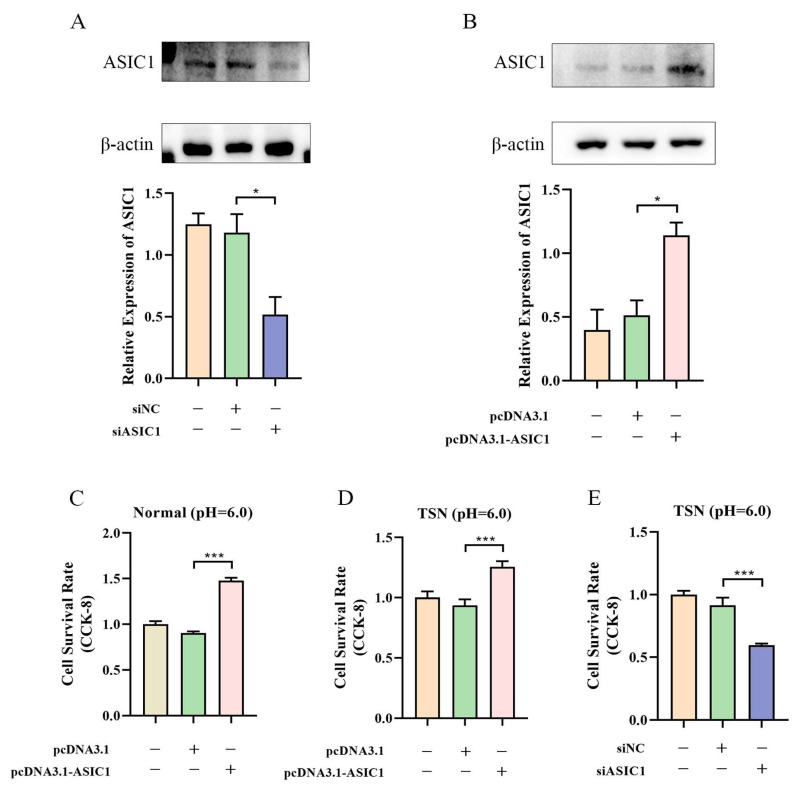
ASIC1 enhances cell survival in the acidic tumor microenvironment. (**A**,**B**) Western blot validation of ASIC1 overexpression and knockdown in HepG2 cells. (**C**,**D**) CCK-8 assay showing that ASIC1 overexpression increases HepG2 cell viability in both pH 6.0 normal medium and pH 6.0 TSN. (**E**) Silencing ASIC1 with siRNA significantly reduces HepG2 cell viability in pH 6.0 TSN. Data are shown as mean ± SEM, *** *p* < 0.001, * *p* < 0.05. In the statistical bar graphs, the orange bars represent the blank control group, the green bars represent the empty-vector control group, the red bars represent the ASIC1-overexpression group, and the purple bars represent the ASIC1-knockdown group. Original images of (**A**,**B**) can be found in Supplementary Materials.

**Figure 4:**
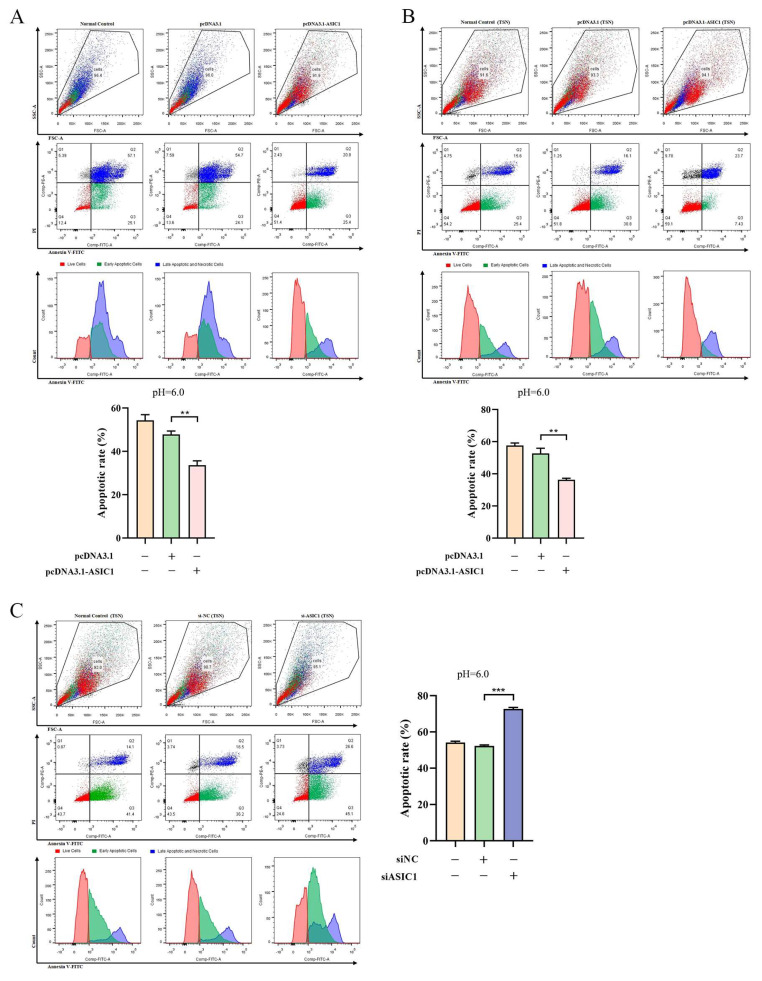
ASIC1 inhibits apoptosis in the acidic tumor microenvironment. (**A**,**B**) Flow cytometry analysis of apoptosis in HepG2 cells cultured in pH 6.0 normal medium and TSN. ASIC1 overexpression reduces apoptosis under acidic conditions. (**C**) Silencing ASIC1 significantly increases apoptosis in pH 6.0 TSN. Data are presented as mean ± SEM from at least three independent experiments, *** *p* < 0.001, ** *p* < 0.01. In the statistical bar graphs, the orange bars represent the blank control group, the green bars rep-resent the empty-vector control group, the red bars represent the ASIC1-overexpression group, and the purple bars represent the ASIC1-knockdown group.

We used an ASIC1 expression vector to overexpress ASIC1 and siRNA to knock down its expression in HepG2 and Li-7 cells. A Western blot confirmed the successful manipulation of ASIC1 expression (Figure 3A,B and Figure S3A,B). Overexpression of ASIC1 significantly increased the viability of HepG2 cells in both pH 6.0 normal medium and pH 6.0 TSN (Figure 3C,D). Conversely, silencing ASIC1 with siRNA in pH 6.0 TSN significantly reduced cell viability (Figure 3E). Flow cytometry analysis showed that ASIC1 overexpression inhibited apoptosis in both pH 6.0 TSN and pH 6.0 normal medium, while silencing ASIC1 in pH 6.0 TSN significantly increased apoptosis (Figure 4A–C and Figure S4A,B). Because knocking down ASIC1 under pH 6.0 in the normal medium caused near-complete cell death, we could not obtain reliable flow cytometry data for that condition. These findings underscore ASIC1′s role in acid tolerance.

### 3.4. ASIC1/PFKM Pathway Mediates Liver Cancer Cell Survival in Tumor Acidic Microenvironment

**Figure 5:**
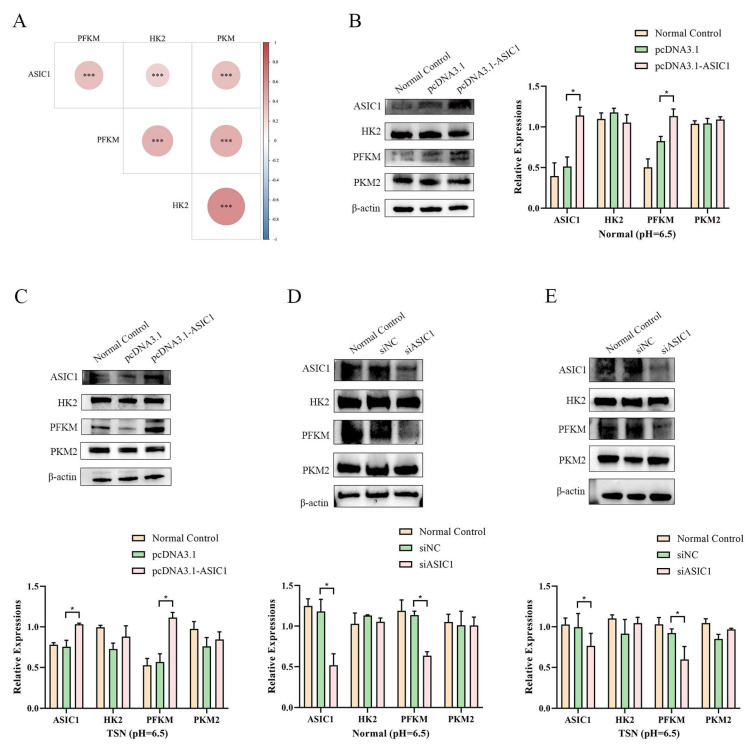
ASIC1 regulates PFKM expression in the acidic tumor microenvironment. (**A**) Correlation analysis using the TCGA database shows a positive correlation between ASIC1 expression and glycolysis-related enzymes, including hexokinase (HK2), phosphofructokinase (PFKM), and pyruvate kinase (PKM2). (**B**,**C**) Western blot analysis shows that overexpression of ASIC1 increases PFKM expression in both pH 6.5 normal medium and TSN. (**D**,**E**) Silencing ASIC1 with siRNA decreases PFKM levels under the same conditions. Data are expressed as mean ± SEM from three independent experiments, *** *p* < 0.001, * *p* < 0.05. Original images of (**B**–**E**) can be found in Supplementary Materials.

During tumor progression, tumor cells predominantly rely on glycolysis to meet their energy demands for unchecked proliferation, thereby gaining a survival advantage in the hostile tumor microenvironment [13]. Analysis of the TCGA database revealed a positive correlation between ASIC1 expression and the gene expression of three glycolysis-related enzymes: hexokinase (HK2), phosphofructokinase (PFKM), and pyruvate kinase (PKM2) (Figure 5A and Table S4). In our study, we transfected an ASIC1 expression vector into HepG2 and Li-7 cells in both pH 6.5 normal medium and TSN. Western blotting confirmed a marked increase in PFKM expression following ASIC1 overexpression (Figure 5B,C and Figure S3A). Conversely, silencing ASIC1 expression using siRNA significantly decreased PFKM levels in both pH 6.5 normal and TSN conditions (Figure 5D,E and Figure S3B). These results indicate that ASIC1 may regulate PFKM expression. We also tested LDH and PFK enzyme activities (Figure S5) in these conditions and found no statistically significant changes, suggesting that additional regulatory factors may influence enzymatic function in this setting.

**Figure 6:**
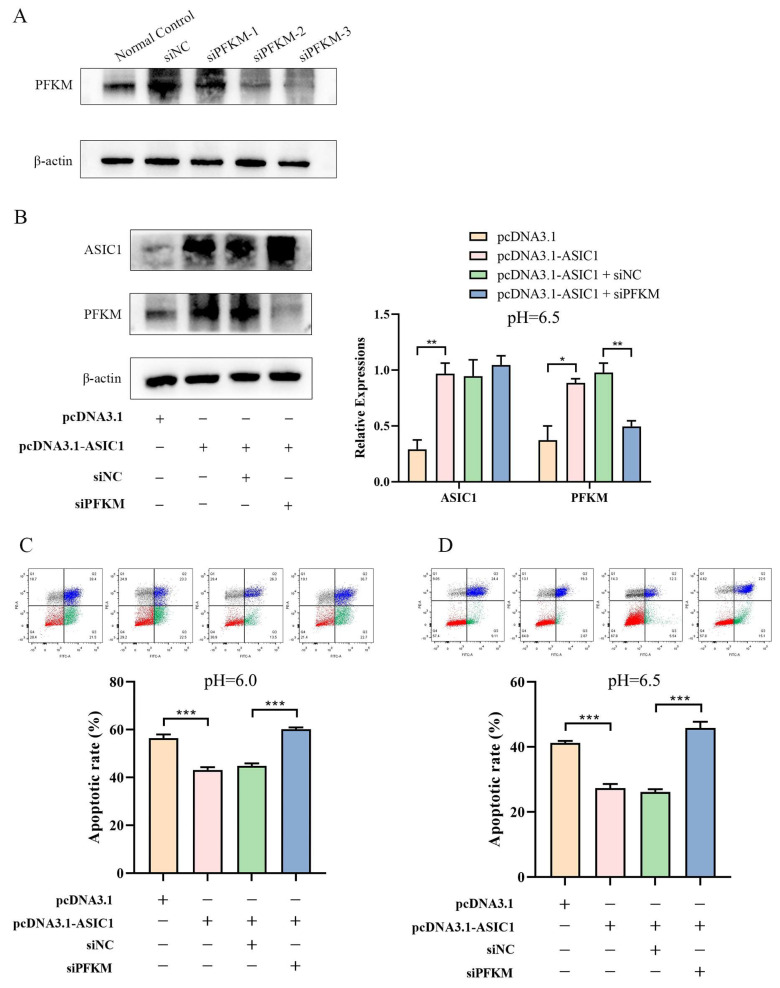
The ASIC1/PFKM pathway mediates HepG2 cell survival in the tumor acidic microenvironment. (**A**) Western blot analysis of PFKM expression in HepG2 cells transfected with three different siRNAs (siPFKM-1, siPFKM-2, siPFKM-3) or negative control (siNC), confirming that siPFKM-3 achieves the most potent knockdown. (**B**) Western blot validation of co-transfection of an ASIC1 overexpression vector (pcDNA3.1-ASIC1) and siPFKM-3 in HepG2 cells grown in pH 6.5 TSN. (**C**,**D**) Flow cytometry analysis of apoptosis in HepG2 cells shows that knocking down PFKM reverses the increased cell viability conferred by ASIC1 overexpression in both pH 6.0 (**C**) and pH 6.5 TSN (**D**). Data are presented as mean ± SEM, *** *p* < 0.001, ** *p* < 0.01, * *p* < 0.05. In the statistical bar graphs, the orange bars represent the empty-vector control group, the red bars represent the ASIC1-overexpression group, the green bars rep-resent the ASIC1-overexpression and siNC group, and the blue bars represent the ASIC1-overexpression and PFKM-knockdown group. Original images of (**A**,**B**) can be found in Supplementary Materials.

To further investigate the role of the ASIC1/PFKM pathway in cell survival, we designed three siRNAs targeting PFKM mRNA (Table S2). Western blot analysis showed that siPFKM-3 exhibited the most potent knockdown effect (Figure 6A). We next co-transfected an ASIC1 overexpression vector and siPFKM-3 into HepG2 and Li-7 cells cultured in pH 6.5 TSN (Figure 6B and Figure S3C). Apoptosis assays revealed that silencing PFKM reversed the increased cell viability caused by ASIC1 overexpression in both pH 6.0 and pH 6.5 TSN (Figure 6C,D and Figure S4C).

## 4. Discussion

Recent studies have shown that the tumor microenvironment becomes progressively more acidic during tumor development, which promotes the malignant behavior of cancer cells [4]. Compared with normal cells, tumor cells exhibit a greater capacity to adapt to this acidified environment. Our findings also demonstrate that an acidic tumor microenvironment promotes the survival of liver cancer cells. To endure these conditions, cancer cells overexpress various pH regulators, including G-protein-coupled receptors (GPCRs) and acid-sensing ion channels (ASICs), enabling them to detect and respond to subtle changes in extracellular pH [24].

ASICs are proton-gated cation channels activated by extracellular acidification, playing essential roles in physiological processes such as learning, memory, and fear conditioning [18]. ASIC1, a subunit of these channels, has been reported to promote the proliferation of gastric cancer, pancreatic cancer, and glioblastoma [21,25]. However, its role and underlying molecular mechanisms in liver cancer cell survival remain unclear. In the present study, we provide evidence that ASIC1 helps maintain cell survival under low pH conditions by regulating PFKM expression and thereby supporting a glycolysis-related survival advantage.

We show a significant upregulation of ASIC1 in liver tumor tissues, correlating with advanced stages and AFP expression. Importantly, this supports the idea that ASIC1 may be involved in more aggressive or advanced disease states [23]. Our cell culture experiments revealed that mild acidification (pH 6.5) increased ASIC1 expression, but viability varied depending on the presence of the tumor supernatant. Under more extreme acidity (pH 6.0), ASIC1 expression was strongly induced in liver cancer cells, conferring a survival advantage that could be abrogated by ASIC1 knockdown. Notably, we validated these observations in both HepG2 and Li-7 cell lines, suggesting that the role of ASIC1 is not restricted to a single model system. Although we are aware that HepG2 is historically classified as a hepatoblastoma line, this cell line has been extensively used in metabolism and acidosis studies related to liver tumors. To address concerns regarding cell line authenticity for HCC research, we performed parallel experiments in Li-7 cells, which yielded results consistent with those in HepG2. Future work may incorporate additional cell lines such as Huh7 or SNU-449 and patient-derived organoids to further validate our conclusions.

The Warburg effect describes the preference of cancer cells to undergo glycolysis rather than oxidative phosphorylation, even in the presence of oxygen. This metabolic shift is thought to provide a survival advantage in hypoxic or nutrient-deprived conditions commonly found in solid tumors. Our study reveals a link between ASIC1 and glycolytic metabolism in liver cancer, as PFKM expression was upregulated following ASIC1 overexpression. PFKM is a rate-limiting isoform of phosphofructokinase, which facilitates the irreversible conversion of fructose-6-phosphate to fructose-1,6-bisphosphate, a key regulatory step in glycolysis [26,27]. Consistent with this mechanism, we observed that knocking down PFKM in ASIC1-overexpressing cells restored apoptosis levels under acidic conditions. This suggests that PFKM mediates, at least in part, the cytoprotective effect of ASIC1 in an acidic microenvironment. While our LDH and PFK activity assays did not show significant changes, possibly due to additional regulatory steps or post-translational modifications, these findings nonetheless reinforce the importance of PFKM in enabling tumor cells to adapt metabolically to acidosis.

In the broader context of the TME, acidosis is only one among several factors, which also include hypoxia, immune infiltration, and nutrient deprivation. It is possible that ASIC1 not only promotes glycolysis-related survival pathways but also impacts immune evasion or response. In future research, we plan to investigate whether high ASIC1 expression in acidified liver tumors modulates immune cell infiltration or T-cell function, which may be highly relevant given the current focus on immunotherapy for advanced liver cancer.

Taken together, our study shows that ASIC1 confers a survival advantage to liver cancer cells under acidic conditions by upregulating PFKM. Although direct glycolytic flux analyses—such as measuring lactate concentrations or conducting Seahorse extracellular flux assays—were not comprehensively performed here, our findings establish a foundation for deeper exploration of the ASIC1/PFKM axis. We propose that targeting ASIC1 or PFKM could offer new therapeutic opportunities by depriving liver cancer cells of their metabolic adaptability to acidic environments.

## 5. Conclusions

In conclusion, we have shown that the acidic tumor microenvironment enhances glycolysis and supports liver cancer cell survival by upregulating PFKM via the acid-sensing ion channel ASIC1. This ASIC1/PFKM axis is pivotal for adapting liver cancer cells to metabolic stress. Our validation in Li-7 cells underscores the broader relevance of these findings. Targeting ASIC1 or PFKM may represent a novel therapeutic strategy for disrupting tumor adaptation in liver malignancies. 

## Data Availability

The datasets in this work are from a public database. All data generated during this study are included in the manuscript and supplementary information.

## Supplementary Materials

The following supporting information can be downloaded at https://www.mdpi.com/article/10.3390/biom15030356/s1: Table S1: Clinicopathologic characteristics of HCC patients; Table S2: siRNAs specifically targeting PFKM mRNA; Table S3: Expression of ASIC1 and AFP in HCC from TCGA database; Table S4: Expression of ASIC1 and three glycolysis-related enzymes in HCC from TCGA database; Figure S1: Spearman correlation between ASIC1 and AFP; Figure S2: Expression of PFKM in different pH conditions; Figure S3: Western blot analysis in Li-7 cells; Figure S4: Flow cytometry analysis of apoptosis in Li-7 cells; Figure S5: LDH and PFK activity assay data. Original images of Figure 2A,F, Figure 3A,B, Figure 5B–E and Figure 6A,B can be found in Supplementary Materials.
