# Supplementary material for: PFKM-Mediated Glycolysis: A Pathway for ASIC1 to Enhance Cell Survival in the Acidic Microenvironment of Liver Cancer
Source: Biomolecules. 2025 Mar 1;15(3):356. doi: 10.3390/biom15030356 (PMC11940220; doi:10.3390/biom15030356)
Supplement: Supplementary file 1 [file biomolecules-15-00356-s001.zip › Table S2. siRNAs specifically targeting PFKM mRNA.pdf]

Table S2. siRNAs specifically targeting PFKM mRNA

ID	Sense	Antisense
siPFKM-1	5’-GCAUCGGAUCAUGGAAAUUdTdT-3’	5’-AAUUUCCAUGAUCCGAUGCdTdT-3’
siPFKM-2	5’-GCUGACACAGCACUCAAUAdTdT-3’	5’-UAUUGAGUGCUGUGUCAGCdTdT-3’
siPFKM-3	5’-GCAGGCAAAUGUUGAACAUdTdT-3’	5’-AUGUUCAACAUUUGCCUGCdTdT-3’
